# Solving teenage and young mothers’ childhood immunization hesitance and non-compliance through mobile immunization friendly service for working mothers in Ibadan, Nigeria- A research note

**DOI:** 10.1371/journal.pgph.0002109

**Published:** 2023-08-03

**Authors:** Mofeyisara O. Omobowale, Olukemi K. Amodu, Folakemi A. Amodu

**Affiliations:** Institute of Child Health, College of Medicine, University of Ibadan, Ibadan, Nigeria; Woolcock Institute of Medical Research, VIET NAM

## Abstract

Mobile Immunization for working mothers (SheVaccs) is an intervention targeted at working mothers in the informal markets of Ibadan to address problem of vaccine hesitance and drop-out among different categories of mother. These mothers have great responsibilities–keeping their homes stable and their children healthy. But these mothers have challenges of different magnitudes that prevented them from immunizing their children, and for teenage mothers they are faced with socio-cultural and socio-economic obstacles and have not responded positively to childhood immunization. In relation to these challenges, SheVaccs intervention study provided friendly immunization, counselling services, and information around vaccination schedules to working mothers in Ibadan, Nigeria. The intervention covered adolescent and young mothers’ population in the selected markets. Mobile clinic was set up in 3 different purposively selected markets in Ibadan. Data were collected through qualitative methods of observation and 21 in-depth interviews with teenage mothers, and 6 key informant interviews with their significant others. All data were subjected to content analysis. The age range of mothers involved in the study was between 17–23 years, almost all participants had no post- secondary school education. All mothers in this study find it difficult to attend conventional immunization centers, due to stigmatization, subtle hostility and embarrassment they experienced during pregnancy and after in some of these centers. Many of them were ignorant and have also been mis-socialized into motherhood and childcare. They preferred an immunization service that is mobile, with “strangers” who are friendly, understanding and will not judge them for ‘‘being anti-social”. Friendly Mobile immunization services targeted at teenage and young mothers will remove clog of stigmatization and hostility and minimize incidence of childhood Immunization Hesitance and non-compliance to schedule.

## Introduction

Immunization is a key cost-effective strategy for the mitigation of vaccine preventable diseases. The global immunization coverage level for children declined from 86% in 2019 to 83% in 2020, with developing nations like Nigeria at the center of this burden [1]. According to World Health Organization (WHO) report, “in 2020 17.1 million infants did not receive an initial dose of DTP vaccine, one of the reasons for this is lack of access to immunization and other health services and an additional 5.6 million are partially vaccinated. Of the 23 million more than 60% of these children live in 10 countries: Angola, Brazil, the Democratic Republic of the Congo, Ethiopia, India, Indonesia, Mexico, Nigeria, Pakistan and the Philippines” [1]. Vaccine preventable diseases are the leading cause of child morbidity and mortality in low-and-middle income countries [2, 3]. However, despite efforts and investments in immunization, vaccination coverages for Nigeria remains low at 31% [4] and incompletion rates are high, although there are regional differences. Several factors have been identified for incomplete and low coverage rates of vaccination generally among nursing mothers [5, 6], but there is dare need to examine subset of mother population across class and cultures in other to contextually deal with barriers and challenges associated with childhood immunization. Examination of subset of mother population have generally shown that young mothers are likely not to have their children completely vaccinated due to individual and contextual factors [7, 8]. These variety of reasons, such as avoidance of unhealthy social relations with the health workforce, as it has been documented by studies that young mothers seeking health services may not treated with adequate dignity by actors in health facilities, as these set of mothers may experience, harassment and embarrassment in words and actions [9–11]. This study through phenomelogical approach philosophy the conceptualization of processes and structure of childhood immunization and how situations (created or natural) are meaningfully lived through as they are experienced, with “nothing added and nothing subtracted” but a pure investigation of what experienced and how experience shape teenage mothers perception and decision about childhood immunization [12, 13].

The intervention study targeted under-five children of mothers on informal work space (the urban market) with mobile immunization clinic with the aim of reducing apathy in childhood immunization coverage among the study population in Ibadan metropolis. This study is intervention research that brought to the fore and further reiterate the importance of adolescent and young people friendly health services as adolescent have been identified as a group of people with specific and different health needs from the general population [14]. In the same vein, bridging immunization gap among adolescent and young mothers also requires a friendly health service for themselves and their children. One of such examples could be childhood vaccination services targeted at adolescent mothers in different regions in line with SDGs number 3 and Agenda 2030 (IA2030) by leaving no one behind in health matters like childhood vaccination. The progress recorded so far increasing demand for vaccination and coverage has been uneven across Nigeria, to this end a Mobile Immunization Clinic (Mobiclic) intervention were set up and operated among working mothers in three purposively selected informal market spaces of Ibadan metropolis, Nigeria. The study therefore identified certain factors associated with immunization hesitance and non-compliance Adolescent and Young Mothers in Ibadan Urban Markets and explore mobile immunization clinic as a possible solution to adolescent and young mothers’ childhood immunization Challenges in Ibadan, Nigeria.

However, examining the above from the prism of cultural response to health issues can be situated within this study, by taking on a brief theoretical explanation. The brief theoretical explanations for this study emanated from PEN-3 model. The model view culture as a collective sense of consciousness with both quantifiable and unquantifiable components, where culture is central to reducing health burden in light of specificity. “The PEN-3 cultural model examines health behavior using a collective approach. The model has been used to assess qualitative data through focus group discussions in studies related to health literacy and adolescent self-care performance for Type 1 Diabetes. The model has also been used to understand HIV/AIDS stigma in the South African family and health care settings and explore the influence of culture on African American mothers’ and daughters’ acceptance of the HPV vaccine” (pp3) [15].

The PEN-3 model was developed in 1989 by Airhihenbuwa [16] to centralize culture in public health and health education programmes in Africa. There are three primary domains in PEN-3 model: relationships and expectations (perceptions, enablers, nurturers), cultural empowerment (positive, existential, negative) and cultural identity (person, extended family, neighbourhood). The PEN-3 emphasizes behaviour within the broader and specific context of culture to distinguish the functions, values and norms that are supportive or not supportive of promoting health activities. PEN-3 offers a cultural lens for addressing health issues and problems like incomplete vaccine uptake among adolescent and young mothers by first identifying the positive aspects of a culture [17, 18].

Furthermore, cultural bahaviours are central to reducing the burden of childhood incomplete vaccination in Nigeria. Adolescent mother’s friendly immunization services deprived of negative perceptions with positive enablers from health workers and nurturers involving family members and older nursing mothers will enhance completion of childhood immunization among adolescent/young mothers. The behaviours of adolescent mothers toward completion of their children immunization are impacted their relationships and expectations, cultural (dis)empowerment and cultural identity, which impact their actions as resources or as a risk factor. For instance, culturally shaped inequality, prevailing cultural norms and values possess a profound influence on the process of stigmatization, labelling, vituperative comments, health/help seeking of adolescent mothers [19, 20].

## Materials and methods

### Ethics statement

The study received approval from Oyo State Research and Ethics Review Committee (AD 13/479/1777B) All participants provided verbal informed consent before the commencement of the interviews, and they were allowed to exit at will. Married teenager below 18 years old are considered emancipated and allowed to take decisions in the study area. Ethical principles in terms of voluntary participation, informed consent, anonymity of the participant’s identity, confidentiality, and no harm to participants, were strictly adhered to all through the study. Ethical approval was obtained before the commencement of the study from Oyo State, Research Ethics Review Committee.

### Methods

Immunization for working mothers (SheVaccs) was intervention research targeted at working mothers in the informal urban markets space of Ibadan. The intervention was done to address problem of vaccine hesitance, zero dose and drop-out incidence among mothers which included adolescent and young mothers. Mothers among the Yoruba of Southwestern Nigeria are saddled with responsibilities/roles of keeping their homes stable and their children healthy, although to many working mothers time is a challenge. In the case of adolescent and young mothers, apart from the time challenge, they are faced with socio-cultural obstacles that debars them from accessing health services like immunization for their children. In response to the identified challenges, Mobiclic provided immunization, counselling services, and information on vaccination schedules to mothers. An exploratory/cross-sectional action research design was adopted. The exploratory approach in this research was informal and unstructured, it also help in the initial understanding of theoretical idea of the research problem [21]. The exploratory method was adopted in order to gain in-depth understanding of the study problem among the working mothers, since there are limited previous studies on immunization and working mothers in Ibadan. The exploratory stance dovetailed into intervention strategies that was repeatedly carried out among working mothers in Ibadan, Nigeria over a ten-month period (June 2020-April 2021).

The intervention was targeted at nursing mothers and also covered adolescent and young mothers’ population in the selected urban markets, namely Oje, Oja-Oba and Bodija markets. These informal economic spaces are composed of a socio-economically disadvantaged population with mostly a traditional family structure. The study was conducted in three purposively (the justification for the selected markets are stated below, as all selected markets were selected based on size, heterogeneity, and large number of working mothers with large socio-economically disadvantage populations, which are key to the study. The study sites and characteristics: 1) Bodija Market a recent, multi-ethnic and modern and largest foodstuff market in the South West of Nigeria. The market population is predominantly female, with numerous adolescents, young, middle aged, mothers and fathers of under-five years children. 2) Oje Market, largely a traditional market which was established over a hundred years ago. The market remains dependent on traditional values and located close to many slum communities of Ibadan. The market is well known for traditional fabrics like aso oke, and it is both daily and periodic market 3) Oja Oba market is one of the oldest traditional markets in Ibadan, it is over one hundred years. The market is located close to the King’s palace and in the midst of traditional (urban) slum communities of Ibadan. It is the hub of politics and commercial transactions of the city. The market accommodates a huge population of working mothers from low socio-economic class across diverse ethnic and sub-ethnic groups in Nigeria.

These markets accommodate a huge population of young mothers that fend livelihood and survival daily on the space. Prior to the commencement of the intervention, the research team embarked on market entry process to identify key leadership and opinion leaders of the study sites, obtained permission and acceptability from the people. This was done in addition to partnership with appropriate governmental bodies at the local cadre in the state. There was commencement of a baseline study with working mothers to generate data that explored knowledge, socio-cultural constructions, experience, perception, understanding and interpretations of working mothers on demand for childhood vaccination, common concerns and barriers to timely and complete childhood immunization. In addition, the baseline study also covered the immunization status of children of mothers in the market and also will be used to identify study participants. The result of the data collected at stage one will be analyzed and use to further strengthen the intervention for working mothers which included adolescent and young mothers. There were also several visitations and enlightenment health talk in many sub-economic group meetings in the selected markets, to create awareness for the intervention. The preliminary data collection/survey earlier mentioned among mothers of under-five in selected markets, to identify suitability of Mobiclic in the markets out of which 3 markets eventually emerged for the study. Mobiclic was set up in 3 different purposively selected markets in Ibadan, it operated once a week per market, for a total of 12 months. In the process of setting up the Mobile Clinic in each market, we identified people who have significant influence on young working mothers. These Social Influencers were able to help reconcile social norms and skepticism to a more pro-vaccination narrative. Respondent for this study were purposefully selected through non-probability sampling. Data were collected through qualitative methods of non-participant observation and 21 in-depth/exit interviews with consenting adolescent and young mothers, and 6 key informant interviews with markets significant others. All participant gave verbal informed consent before the commencement of the interviews, and they were allowed to exit at will. Ethical principles in terms of voluntary participation, informed consent, anonymity of the participants identity, confidentiality, and no harm to participants, were strictly adhered to all through the study. Ethical approval was obtained before the commencement of the study from Oyo State, Research Ethics Review Committee. The interview guide explored the beliefs and attitudes towards immunization, the decision-making process, the perceived barriers, and the enabling factors to access the services. In-depth interviews were transcribed verbatim. Key themes were identified and a coding frame was developed. The comments of the mothers were referenced with the generated themes. Two of researchers who took part in the coding process exchanged the coded material to ensure the reliability. At the end of the in-depth interviews, all the participants children with incomplete immunizations were monitored and encourage in collaboration with Vacc-influencers to complete their vaccinations. All data were subjected to content analysis. The data were processed to established salient themes, patterns and shared experiences of mothers in the study sites. There was transcription and translations of all data by experts to ensure fair and accurate translations of all interviews. In the process of the analysis, all transcripts were read many times and codes generated with focus on key statements, quotes and explanations on childhood immunization experiences of teenage and young mothers. Findings of the study are presented in two main themes with relevant individual extracts.

## Results

The age range of young mothers involved in the intervention was between 17–23 years, almost all participants had no post- secondary school education, a significant number of them only had primary school education. A total number of 21 children of adolescent-young mothers were recruited into the study. At the time they were recruited for the intervention, 7 children were zero dose status and 14 children had drop-out status (in-complete vaccination).

### Identified factors associated with immunization hesitance and non-compliance among children of adolescent and young mothers in Ibadan Urban Markets

#### Stigmatization

Many young mothers find it difficult to attend conventional immunization centers, due to perceived stigmatization, subtle hostility and embarrassment they have experienced in some of these centers. As mentioned by many of them that “The workers there (which sometime may be the ward attendants) are not friendly, they judge us and even abuse me”. The FGD sessions revealed that adolescent mothers hesitate to attend immunization clinic because they feel the clinic environment sees her as promiscuous and disobedient. This view was corroborated by a young mother when she explained reasons behind her reluctant behaviour:

*Well*, *there is nothing*. *The problem is chance to go*, *they waste time and that also affect my business*. *Even my husband used to tell me to go there on Mondays because the center is close to us [her house]*, *they usually vaccinate children on Mondays*, *but sometimes I will lie that I had gone there*. *I just don’t feel like taking my child there again because I believe that its God that can protect my children and those people give me attitude whenever I go there*

**(Young Mother of 3, 22 years, Bodija Market)**.

In addition to the above, the interviews with one of significant others, who was part of the social influencers in the market, one of them lamented that:

*The young mothers are notorious for not completing vaccines for their children*. *I have advised them*, *beg them and even try to report them to older people*, *but they are not yielding*, *they will say the immunization people don’t like them and even sometime insult them and then walk away from immunizing their children*, *but now*, *they seem to prefer this mobile clinic*

**(Social Influencer, 63 years, Oje Market)**

Some adolescent mothers indicated that their hesitation or refusal to go back was because older mothers sometimes give them despicable looks or talk down at the clinics, which discourages them from further attendance.

The vituperative comments that adolescent mothers considered as irritative also come from non-medical staff, as claimed by an adolescent mother in Oja-Oba market that “the matron helper is very toxic, she insulted me and I vowed not to go back, especially when the messenger there also joined her in the abuse that I am still small and should not have a child now”. This also indicate the social conflict between the ideal, socially expected age of motherhood commencement and reality of motherhood in adolescence, thus the production and exchange of vituperative comments which negatively affect immunization uptake.

#### Shared ignorance, mis-socialization and economic inequality

Other factors identified were ignorance, social and economic inequality experienced by mothers sometimes put them in situation where they are unable to afford transportation to health facilities. Distance and economic inequality were identified as obstacle by adolescent and young mothers as reflected in their responses below:

*We did not pay a dime here with you (Mobiclic)*, *where we used to vaccinate*, *they will be abusing us that we came late for vaccination*, *after a long time of begging them*, *they may attend to us but here they did not do that*. *Those that used to go said they pay two hundred*. *I do not pay since I do not go for vaccination again*, *because I cannot afford that every time*

*(* ***Young Mother of 3 children*, *23 years Old*, *Bodija Market*)**

Due to untold economic realities/poverty, experienced by these mothers their view of accessing immunization is impaired as survival take priority on their list, a 19 years old mother said:

*They do not pay me where I work*, *People helps me to feed and feed my children*, *this last one I gave birth to Him in the house*, *because his dad*, *also impregnated another three girls outside*, *I fought him and he decided not to give me money again*, *so how can I go to any immunization center without money for transport and card*. *I am praying that they will be protected*

*(Young Mother*, *19 years Old*, *Oje Market)*

Apart from economic hardship, other factors identified were ignorance, mis-socialization, mis-information, lack of adequate social support, and the experience and the fear of side effects pain displayed by their children after previous vaccination especially after receiving the first dose of Pentavalent vaccine at 6 weeks. These factors were identified as another reason for incomplete vaccination among children of adolescent and young mothers. A late adolescent mother expressed her socialization into ignorance and immunization myth thus:

*It was when people in my community that said I should not immunize my child now*. *When my mother in-law also came*, *she said I must not immunize the baby*. *She said that when she immunized one of her children then*, *the child got ill and sort of*. *She almost lost the child*. *So*, *she told me not to immunize this child*. *I was also told that [by her neighbours] Immunization can make children not to walk on time and can make a child to convulse… I believe it*, *especially when my friend took her baby for immunization [at 6 weeks] and the baby started having high body temperature that she had to rush back to clinic the following day*. *I was scared*

(Adolescent Mother, 19 years Old, Oje market)

Factors associated with hesitance and low vaccine uptake among children of adolescent and young mothers transcends mere age but also include stigmatization, shared ignorance, missocialization/ misinformation into motherhood and childcare as reflected in the above response. Due to such social and environmental factors, childhood immunization coverage will be affected negatively among the study population.

#### Mobile clinic as a possible solution to adolescent and young mothers childhood immunization challenges

The Mobile Clinic was operated from May, 2020 to May, 2021 in 3 urban markets in Ibadan. The markets included Bodija market- the biggest food stuff market in Ibadan and one of its kind in West Africa [22]. Others were Oje and Oja-Oba markets one of the oldest and traditional markets in Ibadan, situated in the traditional slum areas of the city. Adolescent and young mothers preferred an immunization service that is mobile, with “strangers” who are friendly, understanding and will not judge them for ‘‘being anti-social”. In their submission they preferred the Mobile clinic brought to them in the markets because it does not waste time (5 to 7 minutes), which allows them to do other thing or concentrate on their job (source of survival), provide quick, friendly counselling and intervention for their immunization related worries. All young mothers recruited on the intervention were followed for 12 months, completed all basic childhood vaccination, with only one drop out incidence. As reiterated by a significant other whose submission cut across other submissions in the market that:

*The mobile clinic is a good intervention for all these young mothers*, *it has made it easy for us to get them immunize their children*, *we can monitor them*. *Your people are friendly*. *Another good thing is the counseling*. *I have also benefited from it as a grandmother*. *They can concentrate on their business but at the same time also immunize their children*

*(Significant other/Female/ Oja-Oba Market)*

In addition to the above a young mother after experiencing the service from the mobile clinic volunteered to become “advertising agent” for the intervention as revealed in her comment that:

*Now that my child has been vaccinated for free*, *and quickly without wasting time*, *I will tell my friends to come*. *When I see anyone who is yet to vaccinate her child*, *I will tell her to come here*, *you people don’t shame us*. *You are nice and friendly*

*(Young Mother/ Bodija Market)*.

Mobile clinic has also solved the challenge of transport fare and some minor cash related expenses associated with immunization. As pointed out by another adolescent mother that her financial worries related to immunizing her child had been addressed by Mobiclic and desire that such mobile service is replicated across the state and sustained. Her response is paraphrase here:

*I missed my child immunization appointment because I don’t have money to pay for gloves*, *registration*, *even transport to where I will like to take the vaccine*. *I have very little which I will rather use for food*, *but this mobile clinic solved my problem*. *I can now access my child immunization free and without fear*

(Adolescent mother/ 21 years/Oje market).

Some mothers also took advantage of Mobiclic to “secretly” immunize their children, which they have been mis-socialize to immunize. This is more explanatory as a mother lamented that:

I leave with my husband’s people, they have prevented and discouraged me from going to clinic for immunization, they said it is not necessary, but after listening to the health education, I realized I am doing harm to my child, I have immunized him, I will not tell them and if that is what to do to safe my child, I don’t mind

(Young Mother/20 years/ Oja-Oba market)

It is suggestive that friendly Mobile immunization services if targeted at adolescent and young mothers will remove clog of economic inequality, ignorance, misinformation, missocialization, stigmatization, and hostility that prevent adolescent mothers from accessing immunization.

## Discussion

The childhood immunization coverage in Oyo State has not been impressive among mothers generally. It is important that health workers in immunization clinics acquire adequate and non-hostile strategies to communicate with adolescent and young mothers as recommended by Oku et al [23]. It is also important to create a friendly environment that accommodate them in the clinics, all those who work in immunization clinics including lower cadre staff like messenger should be trained on sensitivity of vituperative comments toward adolescent and young mothers, its possible negative effects on immunization coverage and also on how to meaningfully without stigmatization communicate effectively with these mothers. Mobile immunization services targeted at adolescent and young mothers especially those in the low socio-economic circle is the need of the hour for achieving a holistic adolescent and young people friendly health services on the one hand and also to overcome obstacles to childhood vaccination, address effectively vaccine hesitancy and improve childhood vaccination coverage among adolescent and young mothers [24].

Many adolescent and young mothers are faced with other non-health challenges and immunizing their wards does not top their priority list. Due to time challenge and lack of interest whenever they met with obstacle in attempt to immunize their children, they utilize such obstacle as excuse for non-compliance with immunization schedule or for zero dose or incomplete status, this also corroborate the findings of Rahji et al. [25] among mothers in Ibadan Nigeria. This non-compliance attitude may be related to the low educational status of the mothers as out of school/ no/low educational status increases risky behaviour and associated activities like teenage pregnancy, non-compliance to health-related instructions. Maternal education status is important to achieving improved childhood immunization coverage among children of adolescents and young mothers [26, 27]. Intensifying effort in enrolling children in formal educational institution and ensuring reduction in out of school children/adolescent in Nigeria will go a long way to help increase immunization coverage, because many young people will delay child bearing to later age of better maturity, better knowledge and attitude towards health and means of livelihood that can adequately care for themselves and children [28, 29].

Social and economic inequality is entrenched among unskilled adolescent and young mothers within the low-socio-economic class, as indicated in the findings. Many of these adolescent and young mothers are downtrodden and also drop out of school early in life, with no or poor parenting guidance, due to this social inequality and poverty they at early age vulnerable to risky behaviours and they eventually got pregnant to a man that many time fails to take responsibility for them and their child [30, 31]. As rightly pointed out by Masuda and Yamauchi, and Tade [31, 32] many of the husbands of these adolescent and young mothers are “to them, the man is a figurative ‘man’, but not living since what symbolizes being a man such as efficient catering for the family is non-existent…[as these men engage in the paramour act]” (2020: 5). It means there is no complementary production of household needs but a solo provision of needs that is grossly inadequate. To this end mothers in the study are subject of social inequality and patriarchal culture where it is a norm for the “man” to have more than one sexual partner or wives and anti-social for the “women” to engage in multiple sexual partners at once, in light of this these women sort livelihood from the market even though they are unskilled [29–31].

## Conclusion

The study is an intervention to examine possible means of solving adolescent and young Mothers’ childhood Immunization hesitance and non-compliance through Mobile Immunization Clinic service (Mobiclic) for working mothers in Ibadan, Nigeria. The study uncovered relational factors associated with immunization hesitance and non-compliance up-take among children of Adolescent and Young Mothers in Ibadan Urban Markets. Reducing childhood immunization hesitance and non-compliance among adolescent requires friendly childhood immunization services as exemplified with mobile clinic, thus, young mothers in Ibadan require a strategic and friendly immunization service that is mobile, ‘quick’, and friendly. It is also important to prioritize maternal education in the national policy and all parts work together to achieve success. Limited access to education, productive resources, livelihood and the social realities of mis-information and mis-socialization increases hesitancy and non-compliance behaviours among adolescent and young mothers. Mobiclic like friendly immunization service is a possible panacea to immunization hesitance and drop-out among teenage mothers in Ibadan. Addressing non-health factors that are relational, economic, cultural are important determinants to reduce the rate of immunization hesitance and non-compliance among adolescent and young mothers.

### Limitation to the study

The study is limited in its population size, the study is contextual and may not be generalize for a different geographical space.

## Supporting information

S1 Text Interview guide (English). (PDF)

S2 Text Interview guide (Yoruba). (PDF)

S3 Text PRISMA checklist. (PDF)

S1 Data Data set. (RAR)
