# Supplementary material for: Solving teenage and young mothers’ childhood immunization hesitance and non-compliance through mobile immunization friendly service for working mothers in Ibadan, Nigeria- A research note
Source: PLOS Glob Public Health. 2023 Aug 3;3(8):e0002109. doi: 10.1371/journal.pgph.0002109 (PMC10399783; doi:10.1371/journal.pgph.0002109)
Supplement: S1 Text — (PDF) [file pgph.0002109.s001.pdf]

Informed Consent

We are SheVaccs from Institute of Child Health, College of Medicine, University of Ibadan, Nigeria. We are researching on the Immunization Strategies for Working Mothers in Ibadan, Nigeria. We will like to request that you participate in this study by answering the following questions. You are free to opt out at any point when you are not satisfied with the study content.

We therefore if you are willing to participate in the study, kindly sign below.

Participant sign/thumbprint

……………………………

Date………………………

Unstructured Interview guide for IDIs/ KIIs

Probe for:

A. Socio-demographic characteristics

- Age
- No of children;
- Ages of children;
- Gender of children;
- Occupation;
- Income;
- Marital Status ;
- Level of education;
- Type of family;
- Religion;
- Ethnicity;
- Residential pattern
- Technological use- type of phone, ipad, and so on

B. Awareness and knowledge of vaccines/immunization

- Knowledge of immunization
  - Probe the types of vaccine available.
  - The timing of each vaccine.
  - How many vaccines should a child access.
  - When should vaccination begin and end?.
  - What does each vaccine protect against?
  - Knowledge of special vaccines.
  - Do you believe in the effectiveness of vaccines
- How important is , vaccination to you?
  - Compare past and present.

C. Perception of immunization

- To what extent are , vaccines effective? Why do you say so?
- Are there situations in which a child should not be vaccinated?
- Probe for preterm birth, child born by cesarean section, missing dates of appointment , child having fever, level of importance of vaccine
- What is your view about other means of inoculation asides orthodox vaccination?
- Perceived effectiveness of other means immunization
- Why the position
- Give examples of the above with account from experience

- **Socio cultural constructs of immunization**

- What is your community's view about , vaccines?
- Are there norms guiding or against , vaccination? Please explain further

- What are the cultural beliefs surrounding , immunization ?
- How has the view of people about , vaccination changed over time?
- Personal perception of , vaccination

D. Challenges of immunization

- occupation's nature and condition-work schedule
- narrate experiences vaccinating their children
- Describe a typical vaccination day
- Access to facility and availability of vaccines
- How close is the facility to your house, do you walk, if yes, how long? Do you commute by buses or cans or private car.? How long does it take, how much does it cost to get to vaccination facility? What is the estimated cost of transportation to the facility
- If there are other sacrifices/challenges she made in order to vaccinate her child/children- at work, home, personally and so on (with examples)
- Timeliness and completion of the vaccination
- What Time does the facility open? Time she arrives there? When immunization start?
- Financial implications of vaccination
- How much do you have to pay for vaccination, are there other non-financial costs? Do you buy a thing, contribute anything?
- When the first vaccine was given to her child
- Importance of timely vaccination
- Importance of complete vaccination
- Duration of vaccine routine
- Who should bear the burden of vaccination cost- mother/father/relatives/ government

- **Comfortability of health facility**
- What is the sitting arrangement at the health facility, is it comfortable. Is it spacious is there lighting? Are there enough chairs to go round**?**
- Attitude of health care worker
  Are the health care workers friendly? Approachable?
- Waiting time to access vaccination
  How long do you wait before you access service
- Experience of side effect?
  Have your child experienced any side effect after immunization?
  Probe for fever, swellings, others?
- Managing side effect
  How did you manage side effect?

Coping strategies and existing Support system

- Identify coping strategies at home, work, immunization clinic and others
- All support sources available, utilized and experiences over time
- Described with app examples spousal contributions to achieving child immunization

E. Possible remedies to Challenges of immunization

- Describe understanding of mobile clinic
- Perceived benefit of mobile clinic
- How often do you think the clinic should come to your workplace?
- What do you think might make it impossible for the mobile vaccination clinic to work?
- Benefits derived from mobile clinic

- Thank you for your time.
