# Supplementary material for: Solving teenage and young mothers’ childhood immunization hesitance and non-compliance through mobile immunization friendly service for working mothers in Ibadan, Nigeria- A research note
Source: PLOS Glob Public Health. 2023 Aug 3;3(8):e0002109. doi: 10.1371/journal.pgph.0002109 (PMC10399783; doi:10.1371/journal.pgph.0002109)
Supplement: S2 Text — (PDF) [file pgph.0002109.s002.pdf]

ILANA FOR IFI ORO JOMITORO ORO

Awa ni Shevaccs ti o n ri si ilera awon omo kekere ati awon omo lati ogba ile iwe eko giga ti unifasiti Ibadann, Naijera. An se iwadi lori aown ona ti awon obinrin ti on sise ngba lati gba abere ajesara fun awon omo won. A fe ro yin wipe ki e je ikan lara awon ti yio ran wa lowo nipa iwadi yii noipa didaun awon ibere wa. E sile so wipe e ko se mo ni asiko k'asioko ti e ko ba fi aramo awon ibere ti o jeyo nipa iwadi yii.

Ti e ba gba lati kopa ninu iwadi wa, e fi owo si aye ti a pese sile labe iwe yii

Mo fi owo sii_______________

Ojo ati osun _________________

Iwa ti ko si ni sisen tele fun Olojunjoju ati Eleka n jeka

Berelori

(A) Amuye nipa Olusoro

- Ojo ori
- Iye awon ti a bi
- Ojo ori awon omo
- Ako abi abo
- Ise ti won se
- Iye owo ti n wole
- Se ti l'oko
- Bawo ni won se kawe si
- Iru idile wo
- Esin wo
- Eya wo
- Iru idile wo
- Esin wo
- Eya wo
- Iru agbegbe wo ni won gbe
- Iro erop ibanisoro wo ni won lo

(B) Imo nipa abere ajesara fun awon omo kekere
  - Imo niap abere ajesara
  - Bere nipa iru abere ti o wa
  - Bere nipa asiko ti a le gba awon abere yii
  - Abere ajesara melo ni omo gbodo gba
  - Igba wo ni abere ajesara gbodo bere ati pari fun omo
  - Awon aarun wo ni abere kookan n dena
  - Nje won mo nipa awon abere ajeasra Pataki
  - Se e gbabgo ninu ise ti awon abere yii n se
  - Bawo ni abere ajesara fun awon omo ikoko se se Pataki si yin si
  - Eje ki a se agbeyewo ninu ateyinwa ati nisinyi

(C) Irisi nipa abere ajesara fun awon omo kekere
  - Bawo ni abere ajesara se lagbara to, kini idid ti e fi so be?
  - Nje awon idi kan wa ti omo ko se le gba abere ajesara
  - Se iwadi lori awon omo ti osu won ko pe, awon omo ti won fi ise abe bi, pipa ojo je fun abere ajesara ati awon omo ti o ni iba
  - Kini igbagbo yin lori awon ogun ibile ti n dena awon aarun yi yato si abere ajesara

- Irisi nipa igbagbo wipe awon ona miran yato si abere ajesara n sise
- Kini idi ti won fi gbagbo ninu ona yii
- Ese alaye tabi apere lori awon iriri yin nipa awon ona miran yii

(D) Iha ti agbegbe ati asa ko si gbigba abere ajesara fun awon omo kekeke

- Kini igbagbo awon eniyan agbegbe yin nipa abere ajesara
- Kini awon ona to to ati eyi ti ko to nipa abere ajesara ni agbegeb yin
- Nje awon eewo wa ti ko fara mo abere ajesara
- Kini asa ati isese awon eniyan lori abere ajesara
- Se iyipada wa ninu igbagbo awon eniyan lori abere ajesara lati igba de igba
- Kini igbagbo tiyin nipa abere ajesara

(E) Ipenija ti on dojuko awon iya olomo ati gbigba abere ajesara

- Iru ise wo ni won se, ki si ni awon nkan ti o romo ise won
- Ese alaye awon oun ti oju yin ti ri nipa fifun awon omo yin ni abere ajesara
- Ese alaye fun wa ni ojo kan ti e lo gba abere ajesara fun awon omo yin
- Bawo o se rorun fun yin lati gba abere ajesara. Se awon abere na wa nibe?
- Bawo ni ibudo igbabere se sumo yin si, se e ma n fi ese rin ni, ti o ba je be bi iseju melo ni? se e maa n wo oko ni, a bi e maa n wo moto, tabi okada, e lo ni e maa n na ni pato
- Awon ohun wo ni e maa ni fissile ni a I se, tori ki e ele gba abere fun awon omo yin, boya ni ile, tabi ni ibise
- Asiko ati igba wo ni e maa n gba abere. Igbawo ni e si maa n pari gbigba abere yi?
- Igba wo ni ibudo igbabere maa ni si, igba wo ni e maa n debe,
- E lo gan ni pato ni e maa n na lati gba abere yi?
- E lo ni e maa n san fun abere, n je e maa n ra ohunkohun tabi da awon owo kan ni ibudo igbare yi?
- Igba wo ni e gba abere akoko fun omo yin? A
- Awon ere wo ni o wa ninu ki a maa gba abere ni asiko ti o ye fun awon omo wa
- Awon ere wo ni o ninu ki a gba abere pe fun awon omo wa
- Asiko wo, a tip e bawo ni o se ye ki a gba abere yi fun awon omo
- Tani o ye ki o san owo abere awon omo. Se baba ni, iya, ebi tabi ijoba

idera ti o wa ninu ibudo igbabere ajesara

- Bawo ni ijoko yin se ri ni ibudo gbigba abere, se ijoko yi rorun, se o to fun awon iya olomo, se aaye wa dada, n je ina ti e wa nibe
- N je awon osise ibudo yi se yin daada, bawo ni iwa won se ri si yin, se won se ba soro
- Se e maa n duro pe, abi won maa n tete da yin lohun, akoko wo ni e maa n lo ki won to dayin lohun
- Nje abere yi ba awon omo yin lara mu, se ko fa iba ati ara wiwu, ona wo ni e gba kappa isele naa

(F) Ifarada ati awon ikunilapa to wa

- Awon ona wo ni e n gba lati rip e nile, ni ibi-ise, ati ni ibudo igba'bere, ohunkohun ko koja afarada
- Kini awon ohun ti o ran yi lowo lati ri wipe ohunkohun ko di yin lowo. Awon iranlowo wo le mulo tabi awon nkan amayedun

- Kini awon igbese ti oko yin gbe, nipa abere ajesara lati rip e e gba abere ajesara fun omo yin

(E) awon ona ti a fi n ko ju Ipenija to le je yo ninu gbigba abere ajesara fun awon omo

- Salaye ni pa ile iwosan alagbeka
- Ki ni aafani ti e ro pe o le jewo nipa ile iwosan alagbeka
- Bi I igbamelo ni e ro pe won le maa gbe wa fun ayika yin
- Kini awon ohun ti ero'pe o le je idiwo lati ma je ki o se e se

(G) Ifi owo pamo fun abere ajesara: e leyi fun wa ni aanfani lati fi owo diedie pamo fun awon abere ajesara ti o yato

- Kini eronja yin nipa eto yi
- Ki ni aafani ti e ro pe o le wa ninu eto yi
- Elo ni e lee fi pamo fun eto yi
- Elo ni e ro pe e lee fipamo ni ojojumo, ose tabi ni osu
- Kini awon ohun ti ero'pe o le je idiwo lati ma je ki o se e se

(H) Eto agbekale kan lori ero alagbe ka yin

- Ki ni e ro ni pa eto agbekale lori ero alagbeka yin ti yio maa ran yi leti asiko ti o ye ki a gba abere fun awon omo wa
- Ki ni awon aafani ti o rom o eto yi
- Nje e maa fe gba eto naa sori ero alagbeka yin
- Kini awon ohun ti ero'pe o le je idiwo lati ma le gba eto yi si ori ero alagbekayin

E se popu fun asiko yin
